# Clinical and Radiological Evaluation of a Fully Tapered Implant Design Following Immediate Placement with Immediate Provisionalization in the Esthetic Area: A Prospective Case Series Study

**DOI:** 10.3390/dj13110529

**Published:** 2025-11-12

**Authors:** Daniele Cardaropoli, Lorenzo Tamagnone, Alessandro Roffredo, Lorena Gaveglio, Alfonso Alejandro García Huerta

**Affiliations:** 1 Giuseppe Cardaropoli Foundation, corso G.Ferraris 148, 10129 Torino, Italy; lorenagaveglio@virgilio.it; 2 PROED, Institute for Professional Education in Dentistry, 10129 Torino, Italy; archipes@hotmail.com (L.T.); soponnino@gmail.com (A.R.); 3 Private Practice, Morelia 58000, Mexico; perioalex@gmail.com

**Keywords:** immediate placement, immediate restoration, post-extraction socket, bone graft

## Abstract

**Background:** Implant placement after single-tooth extraction in the esthetic zone may be a challenge for clinicians. Immediate placement with immediate restoration may represent a valuable alternative in order to obtain a positive esthetic outcome. In such situations, proper insertion torque represents a clinical need, and fully tapered implants have been shown to be beneficial, as they can provide high primary stability values. The aim of this case study was to assess the esthetic and radiological treatment outcomes following the placement of a fully tapered implant in immediate post-extraction sites with an immediate restoration of single-tooth gap replacement in the esthetic area. **Methods:** A total number of 24 consecutive patients requiring single-tooth implant-supported prosthetic rehabilitation in the esthetic area, presenting an intact site, were selected. All implants were immediately restored using an immediate screw-retained prosthetic rehabilitation. Probing Depth (PD) and Bleeding on Probing (BoP) were assessed for four aspects of the implants and measured at 3 and 12 months, together with Marginal Bone Level (MBL), which was measured at day 0 (baseline) and at 3 and 12 months, while Pink Esthetic Score (PES) was measured at 0 (baseline) and 12 months. **Results:** PD at 3 months was 3.04 mm ± 0.69 mm, while at 12 months it was 2.82 mm ± 0.51 mm. MBL was 2.44 ± 0.64 mm at baseline, 2.25 ± 0.61 mm at 3 months, and 2.16 ± 0.60 mm at 12 months. PES was 11.08 ± 0.86 at baseline and 11.48 ± 0.77 at 12 months. **Conclusions:** Within the limits of the present study, immediate implant placement with immediate restoration seems to be a reliable procedure for replacing single teeth with a proper case selection.

## 1. Introduction

Immediate implant placement (IIP) is a surgical procedure where implant surgery is performed after tooth extraction as part of the same surgical procedure. It has been classified as “Type 1” at the 2004 ITI Consensus Conference. The advantages of this procedure include a reduced number of surgical interventions, a reduction in overall treatment time, and the availability of preexisting bone. Its disadvantages include the site’s morphology, which can complicate optimal positioning and anchorage; a thin biotype, which can compromise optimal results; a potential lack of keratinized mucosa; the fact that additional surgical procedures may be required; and, among other factors, the procedure appears to be technique-sensitive [1].

Not all clinical situations are suitable for immediate implant placement, since an implant should not be placed at the time of tooth removal if the residual bone anatomy precludes the attainment of proper primary stability.

The recent Consensus Conference held by the Giuseppe Cardaropoli Foundation focused on the management of the post-extraction socket. It has been suggested that, in cases of an intact site, immediate implant placement with immediate restoration may represent the optimal choice if proper primary stability can be achieved. Moreover, a bone-to-implant gap of more than 2 mm should be sought and grafted [2]. Primary stability is a key prerequisite in immediate post-extraction implants, since it limits micro-movement and allows for osteogenic cells to adhere to the implant surface, leading to secondary stability (osseointegration) [3]. The factors that influence primary stability are the quality and quantity of bone, surgical techniques, as well as the implant’s design and surface [4]. For this reason, fully tapered implants have been developed in order to improve implant performance by more easily reaching levels of primary stability that are compatible with immediate loading protocols [5,6,7,8].

The straightforward, advanced, and complex (SAC) classification system was developed to aid clinicians in the treatment planning of implant-supported cases. Single-tooth replacement in the esthetic area represents a complex procedure, requiring a team-based approach [9]. It has been suggested that immediate implants may be at a higher risk of failures and complications than delayed implants, since, once an esthetic complication occurs, the restoration of the lost hard and soft tissues to their original presurgical levels is extremely difficult [10], even if the esthetic outcome might be better when placing the implant immediately after tooth extraction [11].

Both the grafting of the bone-to-implant gap [12] and the delivery of an immediate restoration [13] seem to improve the final esthetic outcome, but their actual influence on the changes in soft tissue contours in immediate implants is currently unclear.

The aim of the present clinical study was to evaluate the clinical, radiological, and esthetic parameters of fully tapered implants that were placed in fresh post-extraction sites and immediately restored.

## 2. Materials and Methods

Twenty-four consecutive adult and healthy patients requiring single-tooth extraction in the upper arch, from premolar to premolar, were enrolled in the present prospective single-cohort non-parallel clinical trial. The study population consisted of 13 males and 12 females, mean age 58.5 ± 18.4 years.

The research protocol was approved by the Faculty de Odontologia de la Universitad Michoaca de San Nicolas de Hidalgo, Mexico (08012024). The study was conducted in accordance with Helsinki Declaration of 1975, as revised in 2008. All patients signed an informed consent form.

Local inclusion criteria were the presence of a Class I socket according to Cardaropoli’s classification [14], corresponding to an intact extraction site with favorable anatomical conditions where the buccal cortical bone is intact, there is no gingival recession, and good implant primary stability can be achieved.

Before enrolment, extraction sites were both clinically and radiologically evaluated by performing a 3D Cone Beam examination with a 4 × 4 cm FOV (Hyperion X9pro, Myray, Cefla, Imola, Italy). The reasons for extraction included crown and/or root fracture, endodontic treatment failure, and untreatable caries. Patients with acute periodontal or periapical infections were not included. The systemic exclusion criteria were the existence of metabolic bone disease, current pregnancy, history of radiotherapy or chemotherapy for malignancy in the past 5 years, history of autoimmune disease, and drug consumption that could interfere with implant therapy. Patients who smoked more than 10 cigarettes per day were also excluded, and those who smoked 10 or fewer cigarettes per day were requested to stop smoking for 2 weeks before and after surgery. A comprehensive periodontal examination and professional oral hygiene with scaling and root planing was performed on all patients when needed. Instructions for personal care were delivered to ensure a healthy periodontal environment.

**Figure 1:**
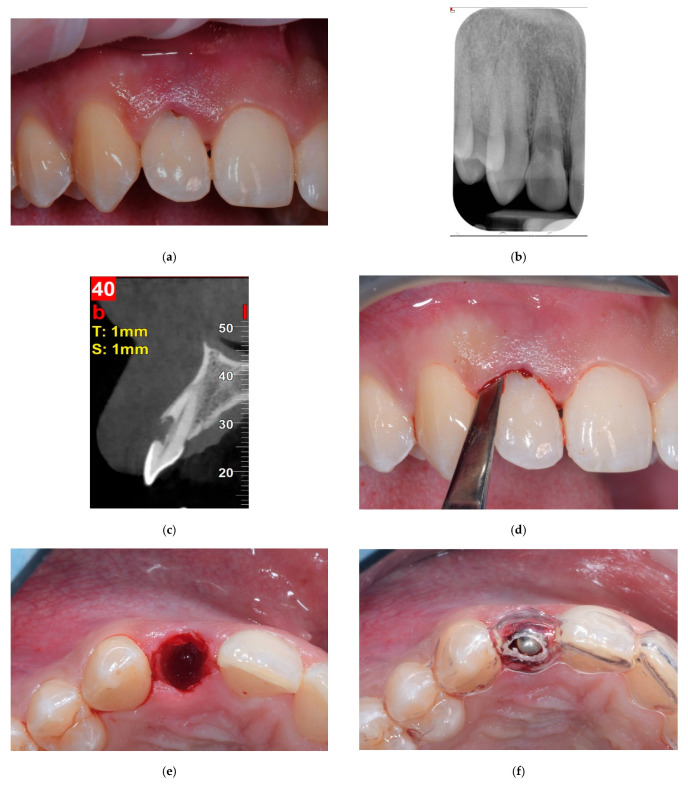

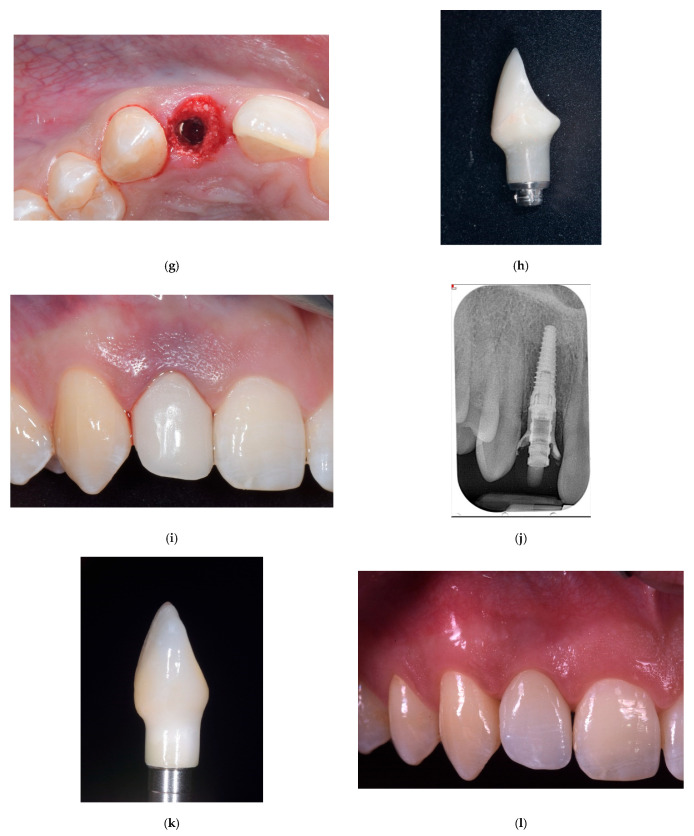

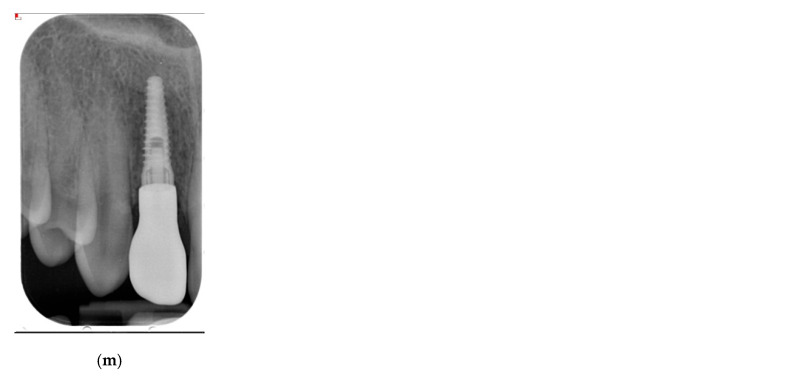
Images from a case of immediate placement with immediate restoration. (**a**) Baseline situation showing an hopeless upper right lateral incisor with a root resorption. (**b**) Baseline intraoral x-ray showing the root resorption. (**c**) Baseline cone beam evaluation, the buccal bone plate is intact. (**d**) The tooth was extracted using dynamic-magnetic approach. (**e**) Due to a flapless minimally invasive approach, soft tissue profile was preserved. (**f**) Implant site preparation was conducted using a prosthetically guided surgical stent. (**g**) A conical edge implant 3.5 mm in diameter and 14 mm in length was inserted slightly palatal, leaving a bone-to-implant gap more than 2 mm pronounced on the buccal side, which was grafted with a bovine bone mineral. (**h**) Chairside, a provisional titanium abutment, was connected and luted to a provisional resin crown. (**i**) The provisional crown was connected to the implant and screwed. (**j**) Intraoral x-ray at the end of the surgery. (**k**) The definitive ceramic crown, three months after surgery. (**l**) Clinical image of the definitive ceramic crown in place, 12 months after implant placement. (**m**) Definitive radiological evaluation, 12 months from implant placement.

Before extraction, in order to provide a prosthetically driven implant positioning, slightly palatal for incisors/canines and centric to the occlusal plane for premolars, a surgical template was fabricated for each patient. The day of surgery, a minimally invasive flapless procedure was performed for tooth extraction. Maximum care was taken to minimize trauma to the socket walls when luxating and extracting the tooth by using a magneto-dynamic approach (Magnetic Mallet, Osseotouch, Gallarate, Italy) (Figure 1a–e). Following extraction, the socket was rinsed with saline solution and the granulation tissue was carefully removed, if present. In order to expose the underlining connective tissue, the epithelium was excised from the sulcus and the junctional epithelium with a 15-C blade. An osteotomy was then performed to prepare for implant placement according to the manufacture’s surgical protocol, using the surgical template as a reference (Figure 1f). In order to underprepare the surgical site and improve implant primary stability, the final drill was used at a low speed (600 rpm). The osteotomy tended to engage a triangle of bone apical and palatal to the apex of the root. The implant platform was placed more than 2 mm from the inner aspect of the buccal bone plate in the horizontal dimension in order to create a wide bone-to-implant gap, and 3.5–4 mm from the expected emergence profile in the vertical dimension, intended as the distance from the implant platform to the free gingival margin.

A total of 24 fully tapered, blasted, and etched implants with highly hydrophilic surfaces (Neoss ProActive Edge, Neoss, Gothenburg, Sweden) were inserted, having diameters of 3.5 mm or 4 mm and lengths of 11 mm, 13 mm, or 15 mm. All implants were engaged reaching a final insertion torque ≥ 35 Ncm.

The bone-to-implant gap was accurately grafted using bovine bone mineral blended with 10% collagen fibers (BioOss Collagen, Geistlich, Wolhusen, Switzerland) (Figure 1g).

A provisional screw-retained abutment (Provisional Ti Abutment, Neoss, Gothenburg, Sweden) was inserted and seated, and a provisional acrylic crown was luted with a light-cured composite resin. The provisional crown was then refined and polished on the chairside. In order to create a fully contoured provisional crown, the composite resin below the free gingival margin was molded with subgingival contours that duplicated the pre-extraction anatomy. The acrylic crown was screwed on the implant at 15 Ncm, and occlusion was adjusted in order to avoid any contact during lateral and protrusive movements and to provide a light infra-occlusion (no contact using the 12 μm paper and contact using the 200 μm paper) (Figure 1h–j). Antibiotic therapy with 1 g amoxicillin plus clavulanate potassium (every 12 h) was prescribed for 6 days. Ibuprofen (600 mg) was also prescribed, but was assumed only on demand. The patients were also asked to use a 0.2% chlorhexidine gluconate mouthrinse every 8 h for 14 days. After 3 months of healing, the immediate provisional restoration was disconnected, a scan body was connected on the implant platform (Scan Body Neoss SP, Neoss, Gothenburg, Sweden), and a digital impression (Trios, 3Shape, Copenaghen, Denmark) was taken. A screw-retained definitive ceramic crown was than created on a titanium base (NeoBase SSC and ASC abutments, Neoss, Gothenburg, Sweden). After 2 more weeks, the screw-retained ceramic crowns were delivered (Figure 1k–m).

### 2.1. Clinical and Radiological Measurements

All measurements were performed by a single person (A.R.) different from the surgeon (D.C.). A blinded examiner (L.T.) evaluated all of the measurements.

Three months (T1) and twelve months (T2) from implant placement, Probing Depth (PD) and Bleeding on Probing (BoP) were measured at the following implant locations: mesial, buccal, distal, and lingual.

Before tooth extraction (T0) and twelve months after implant placement (T2), the Pink Esthetic Score (PES) was evaluated [15]. Briefly, PES evaluates the peri-implant soft tissue around single-tooth implants, and is based on seven variables: mesial papilla, distal papilla, soft-tissue level, soft tissue contour, alveolar process deficiency, and soft tissue color and texture. Each variable is assessed with a 0, 1, or 2 score, with 0 being the poorest and 2 being the best score. The highest possible score is 14. The threshold of clinical acceptability is set at a value of 9 (out of 14).

Three months (T1), six months (T2), and twelve months (T3) from implant placement, Probing Depth (PD) and Bleeding on Probing (BoP) were measured at the following implant locations: mesial, buccal, distal, and lingual.

At implant placement (T0), three months (T1), and twelve months post-loading (T3), standardized digital intraoral radiographs were taken for each implant using the paralleling long-cone technique (Hy-Scan, MyRay, Cefla, Imola, Italy). Radiographs were saved and then acquired and evaluated using ImageJ 1-54i (National Institutes of Health) software. The software was calibrated for every single image using the known length of the implant. Linear measurements on the digital radiographs were performed using the measurement tool specifically designated in the software. Marginal Bone Level (MBL), intended as the distance from the interproximal bone to the reference point on the outer aspect of the implant shoulder at the mesial (mMBL) and distal (dMBL) side, were measured, and their mean values were calculated.

#### Implant Survival and Success

Implants were classified as successful and surviving according to the following definitions:

(a) A surviving implant is an implant that is in place at the time of follow-up.
(b) A successful implant is an implant that meets all of the following criteria [16]: absence of persistent subjective complaints, such as pain, foreign body sensation, and/or dysesthesia; absence of a recurrent peri-implant infection with suppuration; absence of mobility; and absence of a continuous radiolucency around the implant.

### 2.2. Statistical Analysis

A power calculation was performed, determining that a sample size of 20 was necessary to detect a difference in a Marginal Bone Level of 1 mm between baseline and 1-year sites using the paired t-test with 80% power and 0.05 level of significance. Clinical and radiological parameters (PD, BoP, and MBL) were evaluated at baseline, 3 months, and 12 months, with means, standard deviations, and 95% confidence intervals being calculated. PES will be evaluated at baseline and 12 months. All linear measurements were analyzed using the non-parametric statistical method for paired data, and multiple comparisons (Friedman test) and post hoc were analyzed using the Wilcoxon test and Bonferroni correction. Statistical significance was set at *p* < 0.05. All statistics were calculated using SPSS version 25 (IBM) statistical software. In order to compensate any eventual dropout, 24 patients were enrolled.

## 3. Results

All patients completed the study, and all surgical and prosthetic procedures were performed as planned. All implants reached a minimum insertion torque of 45 Ncm, higher than what was set in the inclusion criteria (35 Ncm).

Regarding the safety of the procedure, no adverse events were reported spontaneously by the subjects or observed by the surgeon or their staff. Neither implant nor procedure related adverse events were reported.

All implants osseointegrated and were in function at 1 year from placement/loading, with a 100% survival rate. All implants fulfilled the success criteria, so the success rate was 100%.

Mean Probing Depth (PD) at 3 months was 3.04 ± 0.69 mm, while at 12 months it was 2.82 ± 0.51 mm. The difference was not statistical significant for all sites, except for the lingual sites. Bleeding on Probing (BoP) was 7.00 ± 13.54% at 3 months and 10.00 ± 12.50% at 12 months, with a statistically significant difference (Table 1).

Bleeding on Probing (BoP) was 7.00% ± 13.54% at 3 months and 10.00% ± 12.50% at 12 months (Table 2).

Pink Esthetic Score (PES) was 11.08 ± 0.86 at baseline and 11.48 ± 0.77 at 12 months. The difference was not statistically significant. At this time, the threshold of clinical acceptability (a value of 9) was largely exceeded. Mean Marginal Bone Level (MBL) was 2.44 ± 0.64 mm at baseline, 2.25 ± 0.61 mm at 3 months, and 2.16 ± 0.60 mm at 12 months. All differences were of statistical significance (Table 2).

Mean Marginal Bone Level (MBL) was 2.44 ± 0.64 mm at baseline, 2.25 ± 0.61 mm at 3 months, and 2.16 ± 0.60 mm at 12 months. All differences were of statistical significance. (Table 2).

## 4. Discussion

The present research protocol aimed to evaluate soft tissue contour and bone-level stability in patients undergoing immediate implant placement with immediate provisionalization. In the present study, both success and survival rates were 100% at one year, confirming the outcomes of a systematic review on immediate implants, where a two-year survival rate of over 98% has been reported, with an annual failure rate of less than 1% [17].

Immediate implant placement is beneficial to reduce the overall treatment time, prevent the need for additional surgery, and increase patient comfort [2,18]. Furthermore, immediate placement, when followed by immediate restoration, may eliminate the need for a removable provisional prosthesis and maximize patients satisfaction and esthetic outcomes [2,9,19].

The possibility to keep the external gingival contour stable when placing immediate implant with immediate provisionalization has been clinically described. The latest strategy seems to reduce volume loss and shrinkage area at the external layer when compared to a delayed rehabilitation strategy [20,21]. In the present study, the Pink Esthetic Score was used to evaluate gingival esthetic appearance. The mean PES value was 11.08 at baseline and 11.48 at 12 months, remaining numerically and statistically stable. At both timelines, the threshold of clinical acceptability (a value of 9) was largely exceeded. This indicates that an immediate provisionalization is able to support the pre-existing soft tissue profile and give stability to the gingival architecture.

Management of the post-extractions site can still represent a challenge for clinicians, since the remodeling of the socket—due to the resorption of the bundle bone, a tooth-dependent structure that contains the collagen fibers of the periodontal ligament, and also because of the lack of nutrition—is lost after extraction and is replaced by woven bone [22]. Immediate implant placement alone cannot prevent the remodeling of the post-extraction socket, and marked volume alterations have been reported at the end of the healing period [13,23]. Recently, both a systematic review [24] and a Consensus Conference [2] held by the Giuseppe Cardaropoli Foundation supported the importance an adequate bone–implant gap and the preference for using xenografts as gap fillers.

The improved Marginal Bone Level reported in the present study is characteristic of immediate implants, since the implant platform is initially located sub-crestally and the implant progressively achieves bone-to-implant contact in the coronal portion. The xenograft, which fills the bone-to-implant gap, may also play a role in this process [25].

Probing Depth remained stable during the follow-up period, with a minimal change without clinical significance from T2 (3 months from surgery) to T3 (12 months from surgery). Likewise, Bleeding on Probing never exceeded 15% during the evaluated period, confirming the absence of inflammation in the peri-implant soft tissues.

The results reported in the present study support the use of immediate restoration protocols following immediate placement. The chance to deliver an immediate prosthesis is directly related to the ability to achieve a satisfactory primary stability, normally set at 35 Ncm [2,3,26,27,28].

This clinical protocol is the first ever to test this new fully tapered implant system (Neoss Edge). The results obtained appear to validate its efficacy in very specific contexts, such as immediate insertion with immediate loading.

## 5. Conclusions

Within the limits of the present study (e.g., lack of a control group, short follow-up, the absence of volumetric soft tissue analysis) it can be concluded that when proper primary stability is achieved, immediate implant is a reliable solution for single-tooth replacement. Accurate grafting of the bone-to-implant gap may help in compensating for post-extraction bone remodeling, and immediate restoration is helpful in supporting the original soft tissue contour, providing optimal esthetic outcomes.

## Figures and Tables

**Table 1:** Probing Depth (PD) values at T1 (3 months) and T2 (12 months). Mesial PD (mPD), buccal PD (bPD), distal PD (dPD), lingual PD (lPD), and mean PD (mnPD). Bleeding on Probing values at T1 and T2. *p* < 0.05.

	3 Months	12 Months
**meanPD (mm)**	3.04 ± 0.89	2.82 ± 0.51
**mPD (mm)**	2.90 ± 0.91	2.70 ± 0.57
**bPD (mm)**	2.75 ± 0.72	2.70 ± 0.73
**dPD (mm)**	3.10 ± 1.21	2.75 ± 0.79
**lPD (mm)**	3.25 ± 0.91	3.05 ± 0.76 *
**BoP (%)**	7.00 ± 13.54	10.00 ± 12.50 *

* The difference is statistically significant.

**Table 2:** Marginal Bone Level (MBL) values at T0 (baseline), T1 (3 months), T2 (12 months). Mesial MBL (mMBL), distal MBL (dMBL), mean MBL. *p* < 0.05.

	Baseline	3 Months	12 Months
mMBL (mm)	−2.44 ± 0.71	−2.27 ± 0.67	−2.19 ± 0.64
dMBL (mm)	−2.45 ± 0.64	−2.22 ± 0.60	−2.12 ± 0.58
meanMBL (mm)	−2.44 ± 0.64	−2.25 ± 0.61	−2.16 ± 0.60

## Data Availability

Data may be available upon request to the corresponding author.
